# Internet-Communication Disorder: It's a Matter of Social Aspects, Coping, and Internet-Use Expectancies

**DOI:** 10.3389/fpsyg.2016.01747

**Published:** 2016-11-10

**Authors:** Elisa Wegmann, Matthias Brand

**Affiliations:** ^1^Department of General Psychology: Cognition and Center for Behavioral Addiction Research (CeBAR), University of Duisburg-EssenDuisburg, Germany; ^2^Erwin L. Hahn Institute for Magnetic Resonance ImagingEssen, Germany

**Keywords:** Internet addiction, social networking sites, Internet-use expectancies, psychopathology, personality, coping, online communication

## Abstract

Online communication applications such as Facebook, WhatsApp, and Twitter are some of the most frequently used Internet applications. There is a growing amount of individuals suffering diminished control over their use of online communication applications which leads to diverse negative consequences in offline life. This could be referred to as Internet-communication disorder (ICD). The current study investigates the role of individual characteristics (e.g., psychopathological symptoms, feelings of loneliness) and specific cognitions. In a sample of 485 participants a structural equation model was tested to investigate predictors and mediators which may predict an excessive use. The results emphasize that a higher level of social loneliness and less perceived social support enhance the risk of a pathological use. The effects of psychopathological symptoms (depression and social anxiety) as well as individual characteristics (self-esteem, self-efficacy, and stress vulnerability) on ICD symptoms are mediated by Internet-use expectancies and dysfunctional coping mechanisms. The results illustrate mediation effects which are in line with the theoretical model by Brand et al. (2016). As suggested in the model social aspects seem to be key predictors of ICD symptoms. Further research should investigate convergent and divergent factors of other types of specific Internet-use disorders.

## Introduction

In everyday life, the Internet is an expedient tool to search for information, to shop online and moreover, it serves to communicate with individuals all over the world. The easy access and the increasing use of smartphones enhance the popularity of social networking sites (SNS), such as Facebook, and further communication applications, such as Instagram, Twitter, and WhatsApp (Wu et al., 2013). All these applications enable interaction with other people, effectively interaction is a main characteristic of these tools as part of social media. However, the definition of social media is more extensive: “*Internet-based channels that allow users to opportunistically interact and selectively self-present, either in real-time or asynchronously, with both broad and narrow audiences who derive value from user-generated content and the perception of interaction with others*” (Carr and Hayes, 2015, p. 50). This definition contains key elements such as user-generated value or masspersonal communication, which are also parts of professional network sites, chatboards, or discussion forums (Carr and Hayes, 2015). For this study, we defined Internet communication as the use of social networking sites (e.g., Facebook, Twitter, Instagram), microblogs, and blogs, as well as online messengers (e.g., WhatsApp). Use of these sites involves activities which allow the exchange with other users such as posting content or reading posts. The definition does not include further features of social networking sites such as games or searching for information.

Some of the main reasons these tools have reached such popularity besides the possibility to stay in contact with friends are impression management and to entertain oneself (Krämer and Winter, 2008; Neubaum and Krämer, 2015). Kuss and Griffiths (2011b) detected social factors like group identification and collective's self-esteem as a main predictor for participating in SNS. SNS are web-based communities in which individualized profiles can be created to share personal information and to connect with other users. Online communication applications mainly focus on communication between different persons. In contrast to SNS, social games and information search are not main features of communication applications. (Amichai-Hamburger and Vinitzky, 2010; Kuss and Griffiths, 2011b; Floros and Siomos, 2013; Guedes et al., 2016). However, there is a growing amount of individuals experiencing negative consequences due to excessive use of the Internet or various online applications, such as online communication. This excessive use is referred to as Internet addiction or specific Internet-use disorder. Possible negative consequences could be impaired performance in job, school, or college, conflicts with family and friends, or negative emotions (Brand et al., 2016). The prevalence rate of Internet addiction is reported to be 1% in Germany (Rumpf et al., 2011).

Specific Internet-use disorder describes the addictive use of a certain application, e.g., Internet pornography, Internet gaming, or Internet communication (for an overview see Young, 1998; Young et al., 1999; Griffiths, 2000; Davis, 2001; Kuss and Griffiths, 2011b; Brand et al., 2014b). The addictive use of Internet-communication is often referred to as SNS-addiction, pathological SNS use, as well as Facebook addiction, or smartphone addiction (Griffiths et al., 2014; Ryan et al., 2014; Choi et al., 2015; Wegmann et al., 2015). All these terms apply to the overuse of online communication, social networks, or further Internet-communication services, not of the further specific features such as games in social networking sites (Kuss and Griffiths, 2011b; Casale et al., 2015). Overall, the main aspects of these technologies are the communication and the interaction with others, independent of specific features. Some individuals suffer from negative consequences, such as feelings of loneliness, impaired social activities, psychological health, well-being, or interpersonal relationships, problems with emotion regulation, and limited access to coping strategies, due to the use of these kinds of online applications (Andreassen and Pallesen, 2014; Hormes et al., 2015). In the following the term Internet-communication disorder (ICD) will be applied which is consistent with the DSM-5 terminology of Internet-gaming disorder (American Psychiatric Association, 2013) and furthermore recommended by Brand et al. (2016). Based on the symptoms of behavioral addictions in general and on the classification of Internet-gaming disorder in section III of the DSM-5 in specific, symptoms of ICD are salience, mood modification, tolerance, withdrawal symptoms, loss of control, preoccupation, and negative consequences in job, school, academic performance, or in social relationships (Griffiths et al., 2014).

Brand et al. (2016) suggest a theoretical process model named I-PACE model (I-PACE stands for Interaction of Person-Affect-Cognition-Execution) which addresses potential processes and mechanisms underlying the development and maintenance of a specific Internet-use disorder such as the ICD. This model focuses on the interaction between person's core characteristics, affective and cognitive responses, and the decision to use a certain application. These mechanisms could lead to a gratification and compensation effect possibly resulting in a specific Internet-use disorder. The theoretical framework differentiates between predisposing factors and moderating as well as mediating variables. The authors argue that individuals have certain characteristics, such as personality, social cognitions, specific motives for using an application, psychopathology, and bio-psychological constitution. These characteristics influence affective and cognitive responses such as coping style and Internet-related cognitive biases, for example the Internet-use expectancies. These variables are defined as moderating/mediating variables in the I-PACE model. Internet-use expectancies are defined as the expectations the user has toward the use of the Internet or specific applications. For example, users may expect that using the Internet helps to relief from real-life problems, to avoid loneliness, or to experience pleasure and to gain positive emotions when being online (Brand et al., 2014a). These expectancies can influence one's behavior and the decision to use or not to use a certain application. In the I-PACE model, Brand et al. (2016) assume that especially the effect of the person's characteristics on the development and maintenance of an Internet-use disorder is mediated by coping style and Internet-related cognitive biases. The specific motives and predisposing factors are reinforced by the experienced gratification and the escape from negative feelings. As a result, the excessive use of the preferred application can be increased, resulting in a diminished control and a reduced stabilization of the person's core characteristics (Brand et al., 2016). Some parts of the theoretical process model and its previous version (Brand et al., 2014b) have already been tested empirically with respect to cybersex addiction by Laier and Brand (2014), addictive use of SNS by Wegmann et al. (2015), and generalized Internet addiction by Brand et al. (2014a) using a structural equation modeling approach. The results for generalized Internet addiction showed that coping style and Internet-use expectancies completely mediate the effects of personality and psychopathological aspects on a generalized Internet addiction (Brand et al., 2014a).

Further mediation effects between a person's core characteristics and coping styles as well as Internet-related cognitive biases, which are assumed in the I-PACE model, need to be investigated for the different Internet-use disorders. The current study tested potential predictors and mediators for Internet-communication disorder. Considering the identification of convergent and divergent mechanisms of different types of specific Internet-use disorders the empirical model including the same operationalization as Brand et al. (2014a) was applied to compare direct and indirect mediation effects on a theoretical level.

In the following, the role of certain potential predictors and mediators for maintenance and development of an ICD will be discussed. All predictors we address have been investigated in an earlier study about generalized Internet addiction (Brand et al., 2014a). We also mention further studies, which reveal bivariate or direct effects between the hypothesized predictors and ICD symptoms.

Previous studies for example demonstrated the relationship between ICD symptoms and depression as well as social anxiety (De Cock et al., 2013; Panek et al., 2013; Hong et al., 2014; Bodroza and Jovanovic, 2015; Laconi et al., 2015; Moreau et al., 2015; Guedes et al., 2016). Shyness and low self-esteem have also been linked to ICD symptoms in general or Facebook addiction in particular (Chak and Leung, 2004; Steinfield et al., 2008; Omar and Subramanian, 2013; Panek et al., 2013; Bhagat, 2015; Laconi et al., 2015; Guedes et al., 2016). On the other hand, Jelenchick et al. (2013) found no direct effect between SNS use and symptoms of depression.

Further studies have investigated the central role of loneliness in Internet addiction and ICD. Hardie and Tee (2007) showed that problematic Internet use is associated with high loneliness, social anxiety, and less perceived social support (Hardie and Tee, 2007). Kim et al. (2009) argued that lonely people compensate deficits in real-life when being online. This is in line with studies in which a relationship between loneliness and ICD was found (Baker and Oswald, 2010; De Cock et al., 2013; Omar and Subramanian, 2013; Song et al., 2014). Baker and Oswald (2010) explained that the environment of online communication applications seems like a safe surrounding for shy people who are then enabled to interact with other individuals. This may be particularly relevant if less social support and high loneliness is perceived. It seems that the use of SNS could reduce loneliness, which leads to an increasing Internet use to gratify the need of social interactions (Song et al., 2014). The results emphasize that rather social loneliness than emotional loneliness enhances the use of online communication (Ryan and Xenos, 2011; Jin, 2013). Overall, all of these studies investigate the direct effect between the person's characteristics and the pathological use of different communication applications. However, potential mediation effects by coping style or Internet-related cognitive bias, which are postulated in the theoretical approach by Brand et al. (2016), have not been investigated so far. Merely Wegmann et al. (2015) showed that the effect of psychopathological symptoms, such as depression and social anxiety, on the addictive use of SNS was mediated by Internet-use expectancies. This is in line with Hormes et al. (2015) who theoretically argue, that maladaptive SNS use is effected by different reinforcement mechanisms (see also Kuss and Griffiths, 2011b).

As far as we can tell, there are only a few studies that investigated the role of self-efficacy and the use of SNS. In their study, Wang J.-L. et al. (2015) showed that Internet self-efficacy was a significant predictor of SNS use concerning the motivation for SNS use as social and recreational functions. This is consistent with Gangadharbatla (2008) who indicates that Internet self-efficacy has a positive effect on attitudes toward SNS. The relationship between general self-efficacy and ICD has not been investigated so far.

Summarized, there are many studies regarding the relationship between psychopathological symptoms, self-esteem or loneliness and a pathological use of Internet-communication. Previous research about stress vulnerability or self-efficacy as predictors of an ICD, for example, have not been found. Nevertheless, in the current study same predictors were used which also contain stress vulnerability and self-efficacy in the structural equation model in order to be as close as possible to the original model by Brand et al. (2014a). This procedure allows to compare direct and indirect effects of an ICD with the effects already found in a generalized Internet addiction.

On a theoretical level it could be assumed that individuals who suffer from depression and interpersonal sensitivity have the expectancies toward the Internet to feel better or escape from real-life problems. These individuals may also cope with problems by denial or substance use. It is part of a dysfunctional coping strategy. We hypothesize similar effects for individuals with low self-esteem, low self-efficacy, and high stress-vulnerability as well as individuals who feel lonely and perceive less social support. These social and personality aspects could lead to high expectancies that the Internet is a helpful tool to escape from negative feelings or to experience pleasure and fun, when being online. It could be also hypothesized that these characteristics lead to dysfunctional coping strategies as well. Individuals may denial their low self-esteem or ignore feelings of less perceived support instead of tackle with it. All these strategies to handle problematic predispositions could result in specific cognitions which neglect conflict or negative emotions. Then, we assumed that individuals with the expectancies and the idea to solve problems online could lead to an uncontrolled use of online communication applications.

**Figure 1:**
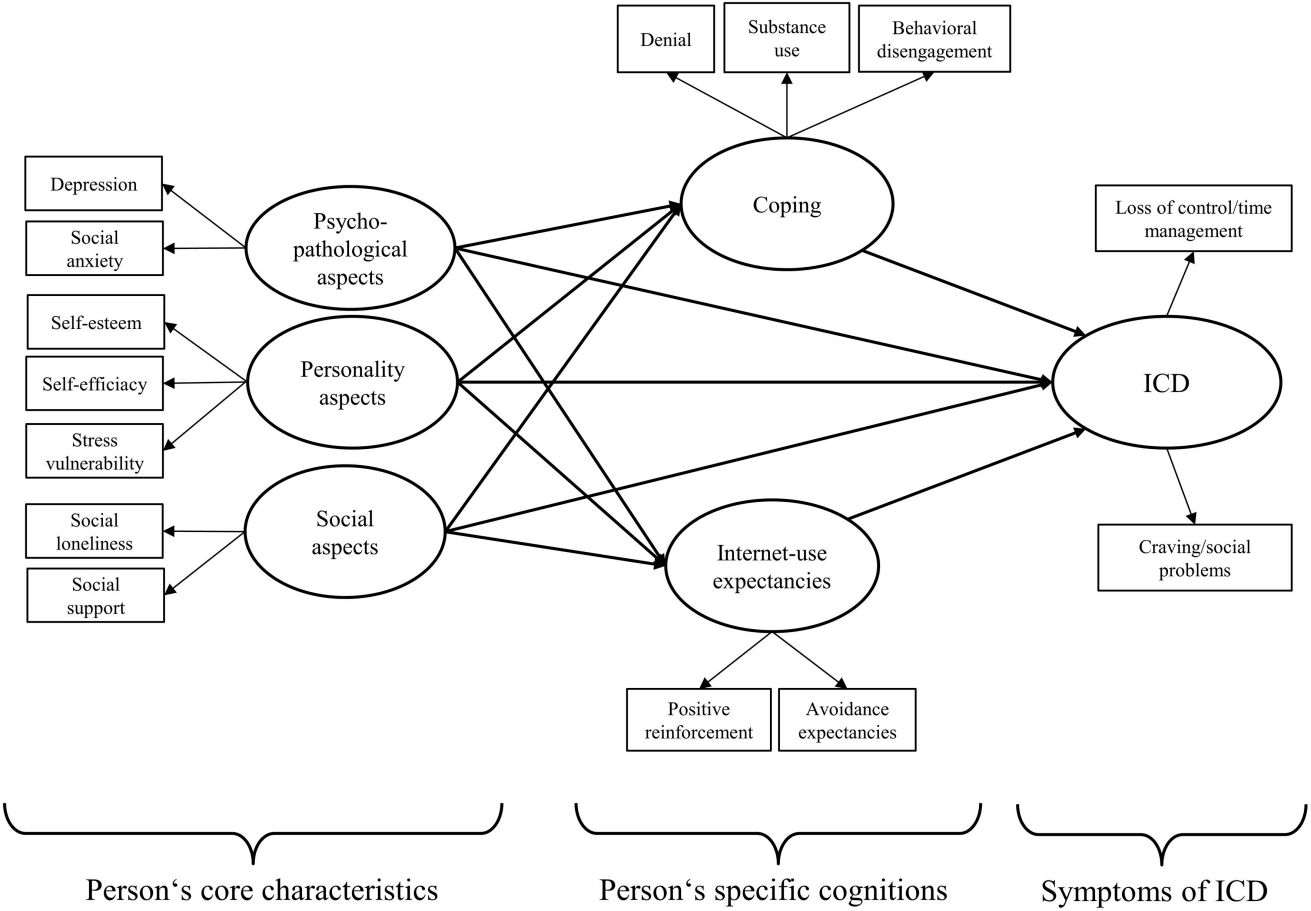
**The operationalized model for analyzing the main assumptions including the latent variables of ICD**.

These considerations are based on the theoretical model by Brand et al. (2016) which mentions these predictors (psychopathological symptoms, personality aspects) are mediated by dysfunctional coping style and Internet-related cognitions such as Internet-use expectancies. Given the literature on the importance of social cognitions for SNS use as postulated by Brand et al. (2016), we argue that the effect of social cognitions on ICD symptoms is only partially mediated by coping style and expectancies. The operationalized model is shown in Figure 1.

## Method

### Participants

Four hundred eighty-five participants aged between 14 and 55 years (*M* = 23.95, *SD* = 4.96 years) took part in the study. Three hundred fifty-eight were females, 125 were males, and two gave no information on gender. Regarding other relevant sociodemographic information, 252 participants reported that they were in a relationship or were married, 366 were students, 115 had a regular job. All participants have participated before in the study of Brand et al. (2014a), in which a sample of 1019 participants was used to test the structural equation model on generalized Internet addiction. The current sample was selected on the basis of the participants first-choice Internet use. We asked the participants to select the specific online application they personally use and that they find most attractive. After the decision was made, participants administered the one version of the short Internet Addiction Test that was specific for their first-choice application. We only included participants who used the Internet mainly for online communication. The analyses using Internet-communication disorder as dependent variable were not part of the former study by Brand et al. (2014a). The participants spend in average 562.10 min (*SD* = 709.03) per week using online communication applications. The sample was recruited at the University of Duisburg-Essen via mailing-lists, flyers, and word-of-mouth recommendations. The assessment was done by an online-survey and the participants could take part in raffle where they have the chance to win an iPad, iPad mini, iPod nano, iPod shuffle, or Amazon gift cards. The local ethics committee approved the study.

### Instruments

#### Modified version of the short internet addiction test (s-IAT-com)

Symptoms of the pathological use of online communication applications like SNS or blogs were assessed with a modified version of the short Internet addiction Test, specified for online communication (s-IAT-com; Wegmann et al., 2015). To assess subjective complaints in everyday life due to online communication applications, the term “Internet” in the original version was replaced by “online communication sites” in all items. The instruction included a definition of online communication, which explained that the term online communication sites includes SNS, blogs, and microblogs, email, and messaging. In the s-IAT-com, participants have to answer 12 items (for example: “*How often do you find that you stay on Internet communication sites longer than you intended*?”) on a five-point-Likert-scale ranging from 1 (=never) to 5 (=very often). Based on the research of Pawlikowski et al. (2013) the sum score ranges from 12 to 60. Within this range, a score >30 indicates problematic use and a score >37 indicates pathological use of online communication applications. The s-IAT-com consists of two factors: loss of control (six items) and craving/social problems (six items). The scale has a high internal consistency (Cronbach's α). For the whole scale α was 0.861 (loss of control/time management α = 0.842, craving/social problems α = 0.774). The scale was used to represent the latent dimension Internet-communication disorder.

#### Internet-use expectancies scale

The Internet-use expectancies Scale (IUES; Brand et al., 2014a) was used to assess the core motivations of participants for using Internet or being online. The questionnaire assesses a general expectancy toward Internet use as a helpful tool for experiencing pleasure or for escaping from reality. Wegmann et al. (2015) already emphasized this scale as a potential factor of an addictive use of SNS. The questionnaire consists of two subscales: positive reinforcement (four items, for example: “*I use the Internet, because it makes it possible/facilitates to experience pleasure”*) and avoidance expectancies (four items, for example: “*I use the Internet, because it makes it possible/facilitates to distract from problems”*). Answers have to be given on a six-point-Likert scale ranging from 1 (= completely disagree) to 6 (=completely agree). In the current sample, internal consistency of positive reinforcement was α = 0.775, of avoidance expectancies α = 0.745. Both manifest variables represented the latent dimension Internet-use expectancies. For a more detailed description see Brand et al. (2014a).

#### Brief COPE

The Brief COPE (Carver, 1997) was used to assess coping style in several subdomains. For the current study, we used three subscales of the German version (Knoll et al., 2005): denial (for example: “*I've been saying to myself ‘this isn't real’.”*), substance use (for example: “*I've been using alcohol or other drugs to make myself feel better”*), and behavioral disengagement (for example: “*I've been giving up trying to deal with it”).* Each subscale consists of two items, which have to be answered on a four-point-Likert scale from 1 (= I haven't been doing this at all) to 4 (= I've been doing this a lot). Internal consistency was for the subscale denial α = 0.495, subscale substance use α = 0.883, and subscale behavioral disengagement α = 0.548, which is mostly comparable with Carver (1997). We consider that the reliability was acceptable given that the subscales consist only of two items and that there are several validation studies including retest reliability (Brand et al., 2014a). The three mentioned subscale were used to represent the latent dimension coping.

#### Brief symptom inventory

The Brief Symptom Inventory was used to assess the psychological status of the participants by self-report (BSI; Derogatis, 1993). We used the two subscales depression (six items, for example: “*In the last 7 days, how much did you suffer from feeling no interest in things.”*) and interpersonal sensitivity (four items, for example: “*In the last 7 days, how much did you suffer from feeling inferior to others.”*) of the German version (Franke, 2000). The answers have to be given on a five-point-Likert-scale ranging from 0 (= not at all) to 4 (= extremely). Internal consistency in our sample was α = 0.863 (subscale depression) and α = 0.798 (subscale interpersonal sensitivity). The latent dimension of psychopathological symptoms was represented by both subscales.

#### Self-esteem scale

To assess self-esteem, we used the modified Self-Esteem Scale by Collani and Herzberg (2003) based on the original scale by Rosenberg (1965). It consists of ten items (for example: “*I take a positive attitude toward myself*.”), which have to be answered on a four-point-Likert scale ranging from 0 (=strongly disagree) to 3 (=strongly agree). Internal consistency was α = 0.904.

#### Self-efficacy scale

An overall self-efficacy was assessed by the Self-Efficacy Scale (Schwarzer and Jerusalem, 1995) consisting on ten items (for example: “*I can usually handle whatever comes my way.”*). Participants respond on a four-point-Likert-scale from 1 (= not true) to 4 (= not exactly true). Internal consistency was α = 0.860.

#### Trier inventory for chronic stress

We measured stress vulnerability in the last 3 months with the Trier Inventory for Chronic Stress (TICS) by Schulz et al. (2004). Twelve items (for example: “*Fear that something unpleasant will happen.”*) have to be rated on a five-point-Likert scale ranging from 0 (= never) to 4 (= very often). Internal consistency was α = 0.910.

The manifest variables of the Self-Esteem Scale, Self-Efficacy-Scale, and the Trier Inventory for Chronic Stress represented the latent dimension personality aspects.

#### Loneliness scale

We used the short version of the Loneliness Scale (De Jong Gierveld and Van Tilburg, 2006) to measure feeling of loneliness. This questionnaire contains two subscales: *emotional loneliness* (three items, for example: “*I experience a general sense of emptiness.”*) and *social loneliness*/*perceived social support* (three items, for example: “*I miss having people around.”*). In the current study we focused on *social loneliness*/*perceived social support*. In this subscale the items have to be rated on a five-point-Likert scale from 1 (= no!) to 5 (= yes!). Internal consistency for *emotional loneliness* was α = 0.755 and for *social loneliness*/*perceived social support* α = 0.865.

#### Social support questionnaire

We measured perceived social support with the Social Support Questionnaire (F-SozU; Fydrich et al., 2009) consisting of 14 items (for example: “*I have a close friend who is always willing to help me.”*), which have to be rated on a five-point Likert scale from 1 (= not true) to (5 = absolutely true). Internal consistency was α = 0.924.

The manifest variable for social loneliness of the Loneliness Scale and the mean score of the Social Support Questionnaire represented the latent dimension social aspects.

### Statistical analyses

The statistical analyses were carried out using SPSS 23.0 for Windows (IBM SPSS Statistics, released 2014). To test bivariate relationships between two variables we calculated Pearson correlations. The confirmatory factor analysis (CFA) and structural equation model (SEM) analyses were computed with Mplus 6 (Muthén and Muthén, 2011). There were no missing data. We evaluated the model fit with the standard criteria: standardized root mean square residual (SRMR; values < 0.08 indicate a good fit with the data), comparative fit indices (CFI/TLI; values >0.90 indicate an acceptable and >0.95 an good fit with the data), and root mean square error of approximation (RMSEA; values < 0.08 indicate a good and 0.08–0.10 an acceptable model fit) (Hu and Bentler, 1995, 1999). The χ^2^ test was used to check, if the data derivate from the defined model. To contrast different models, we considered the Bayesian Information Criterion (BIC) while values lower ten points indicate a better fit with the data (Kass and Raftery, 1995). All relevant variables for the mediation were required to correlate with each other (Baron and Kenny, 1986).

## Results

### Description and correlations

The sample's mean score in the s-IAT-com and the scores of the questionnaires applied and the bivariate correlations can be found in Table 1. In comparison with the reported cut-off scores by Pawlikowski et al. (2013) 39 participants (8.04%) indicated a problematic but not pathological use (cut-off scores >30 but ≤37) and 15 participants (3.09%) a pathological use (cut-off scores >37) of online communication activities.

**Table 1:** **Descriptive statistics and bivariate correlations between the scores of the short Internet Addiction Test and the applied scales**.

**Manifest variables**	**Latent dimension**	***M (SD)***	**2**	**3**	**4**	**5**	**6**	**7**	**8**	**9**	**10**	**11**	**12**	**13**	**14**	**15**
1. s-IAT-communication sum score		22.54 (6.62)	0.924^**^	0.822^**^	0.302^**^	0.484^**^	0.157^**^	0.208^**^	0.253^**^	0.346^**^	0.368^**^	−0.285^**^	−0.236^**^	0.402^**^	−0.252^**^	−0.249^**^
2. s-IAT-com 1	ICD	13.66 (4.49)		0.542^**^	0.264^**^	0.440^**^	0.121^**^	0.152^**^	0.207^**^	0.288^**^	0.280^**^	−0.243^**^	−0.200^**^	0.380^**^	−0.164^**^	−0.144^**^
3. s-IAT-com 2		8.89 (3.01)			0.271^**^	0.408^**^	0.166^**^	0.232^**^	0.247^**^	0.331^**^	0.391^**^	−0.264^**^	−0.221^**^	0.318^**^	−0.310^**^	−0.332^**^
4. IUES – positive reinforcement	Internet-use expectancies	3.80 (0.86)				0.432^**^	0.048	0.056	0.112^*^	0.161^**^	0.169^**^	−0.136^**^	−0.117^**^	0.132^**^	−0.116^*^	−0.122^**^
5. IUES – avoidance expectancies		2.73 (1.03)					0.124^**^	0.171^**^	0.143^**^	0.441^**^	0.433^**^	−0.383^**^	−0.271^**^	0.404^**^	−0.269^**^	−0.293^**^
6. Brief Cope – denial	Coping	1.53 (0.59)						0.191^**^	0.355^**^	0.234^**^	0.197^**^	−0.253^**^	−0.216^**^	0.290^**^	−0.063	−0.115^*^
7. Brief Cope – substance use		1.39 (0.64)							0.136^**^	0.240^**^	0.178^**^	−0.157^**^	−0.088	0.197^**^	−0.105^*^	−0.111^*^
8. Brief Cope – behavioral disengagement		1.42 (0.51)								0.284^**^	0.220^**^	−0.253^**^	−0.249^**^	0.262^**^	−0.169^**^	−0.206^**^
9. BSI – depression	Psychopathological aspects	0.70 (0.73)									0.751^**^	−0.656^**^	−0.467^**^	0.565^**^	−0.434^**^	−0.442^**^
10. BSI – social anxiety		0.92 (0.82)										−0.591^**^	−0.447^**^	0.579^**^	−0.425^**^	−0.411^**^
11. SES – self-esteem	Personality aspects	2.16 (0.54)											0.644^**^	−0.558^**^	0.436^**^	0.444^**^
11. GSE – self-efficacy		2.91 (0.41)												−0.475^**^	0.334^**^	0.377^**^
12. TICS – stress vulnerability		1.75 (0.74)													−0.282^**^	−0.314^**^
13. Loneliness – social	Social aspects	4.11 (0.86)														0.752^**^
14. F-SozU – perceived social support		4.30 (0.64)														

* *p ≤ 0.050*.

** *p ≤ 0.010*.

### Structural equation model

The proposed structural equation model on latent variable with ICD symptoms (s-IAT-com) as dependent variable showed a good fit with the data. The RMSEA was 0.060 (*p* = 0.054), CFI was 0.957, TLI was 0.938, and the SRMR was 0.040, BIC was 15072.15. The χ^2^—test was significant, χ^2^ was 174.17 (*p* < 0.001) and χ^2^/df was 2.76.

**Figure 2:**
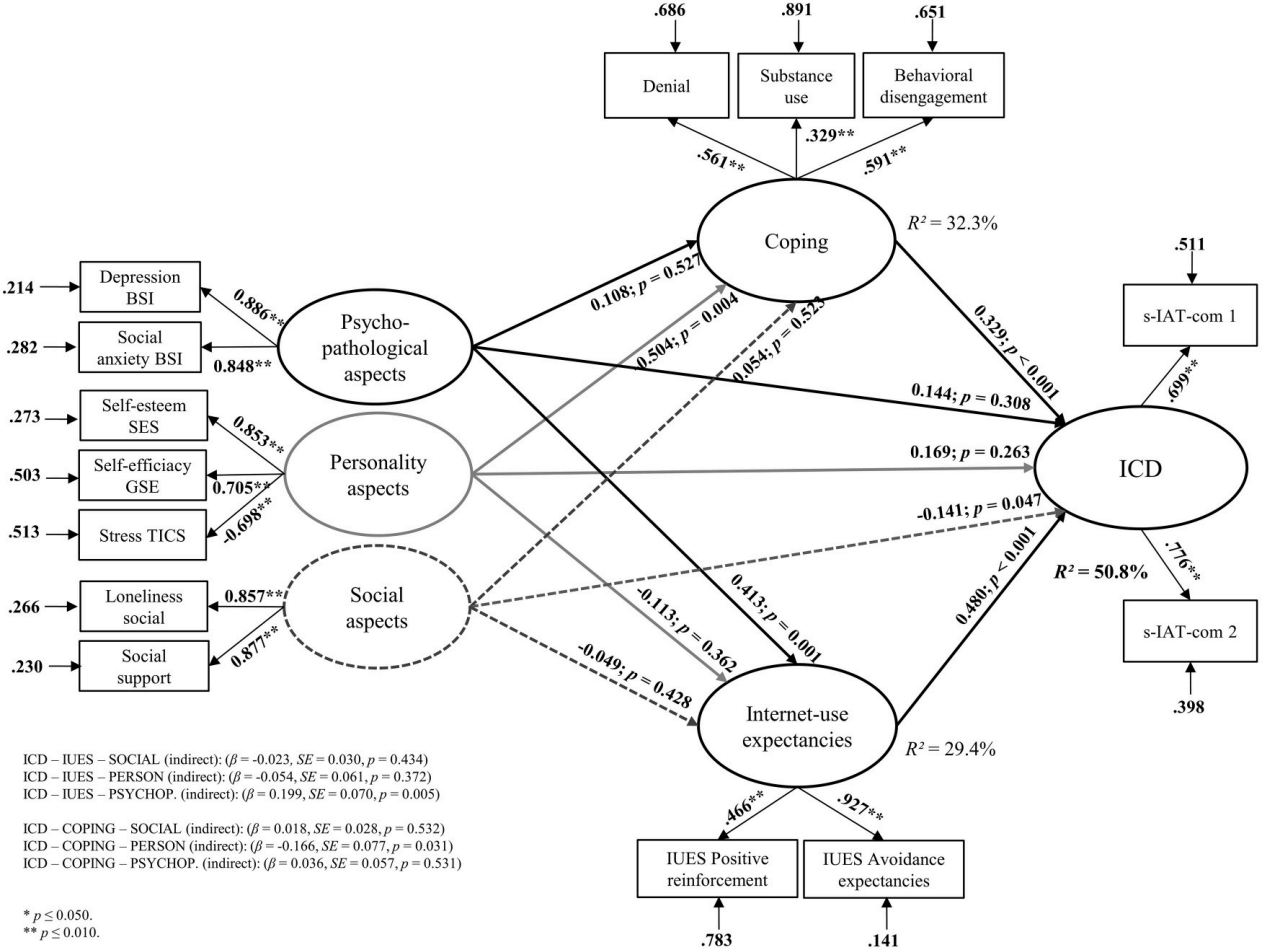
**Results of the structural equation model including factor loadings on the described latent variables and the accompanying β-weights, *p*-values, and residuals**.

Overall, 50.8% of the variance in the ICD symptoms could be explained by the proposed model (*R*^2^ = 0.508, *p* < 0.001). The structural equation model with the factor loadings and β-weights are represented in Figure 2.

The latent variable social aspects had a direct effect on the dependent latent variable ICD while the other latent variables showed no direct effect (all β's < 0.169, all *p*'s > 0.263). However, both mediator variables Internet-use expectancies and coping were significant predictors of ICD. In addition, personality aspects were a significant predictor of coping with a negative β-weight. The indirect effect from personality aspects over coping to ICD was significant (β = −0.166, *SE* = 0.077, *p* = 0.031). The indirect effect from psychopathological symptoms to ICD symptoms over Internet-use expectancies was also significant (β = 0.199, *SE* = 0.070, *p* = 0.005). Both results indicated mediation effects.

### Additional analyses

To better understand further underlying mechanisms of ICD some additional models or parts of the model were tested.

**Figure 3:**
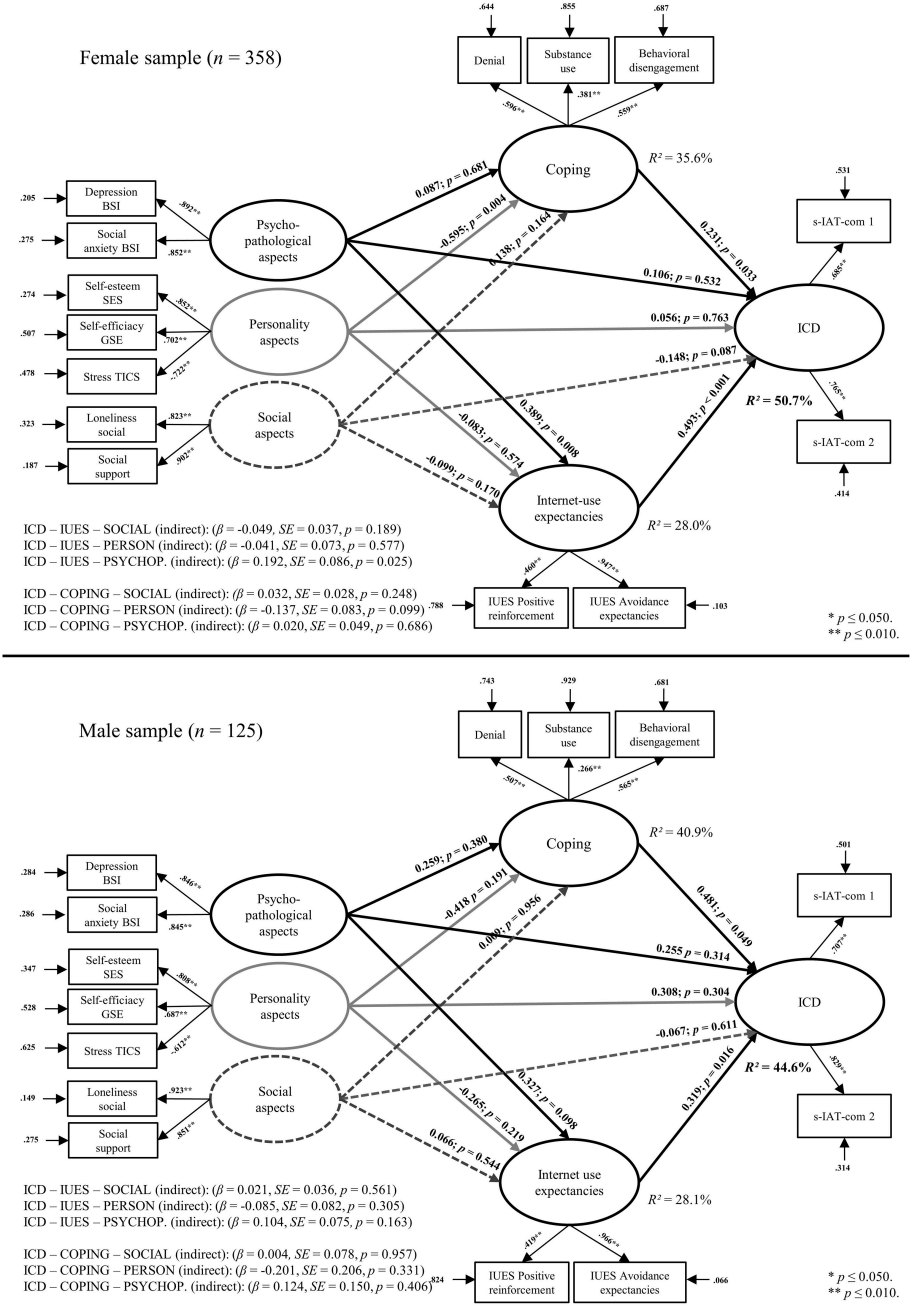
**Results of the structural equation model separated for the female and male sample including factor loadings on the described latent variables and the accompanying β-weights, *p*-values, and residuals**.

The first issue we addressed was the effect of the social aspects on ICD. Compared with the empirical model by Brand et al. (2014a), the latent variable social aspects were conceptualized with the manifest variables *perceived social support* and the latent variable *social loneliness* of the Loneliness Scale by De Jong Gierveld and Van Tilburg (2006) instead of the subscale *emotional loneliness* in the current study. When using the same manifest variables for the latent variable *social aspects*, as done in Brand et al. (2014a), there was an acceptable model fit (CFI = 0.955, TLI = 0.936, RMSEA 0.063, SRMR = 0.040, BIC = 15142.03). However, the difference between this model and the main model of the current study is that there was no direct effect of social aspects or mediation effect of personality aspects and ICD by coping. Demographic variables were also considered as potential variables which may have an effect on the structural equation model. We first calculated bivariate correlations between the manifest variables and age and found only correlations with low effect size (Cohen, 1988) between age and self-esteem, self-efficacy, stress vulnerability, coping variables, and Internet-use expectancies (*r*'s < |0.212|). Overall, the requirements for integrating age in the proposed model were not fulfilled (Baron and Kenny, 1986). To control for gender biases, a group comparison was calculated with all variables and significant differences between male and female participants were found in respect of interpersonal sensitivity, self-efficacy, stress vulnerability, coping subscale substance use, and both Internet-use expectancies factors (*t* = |0.06–4.32|, *p* = 0.035– < 0.001). After this, a structural equation model with additional differentiation by gender using a mean structure analysis was analyzed. This way of proceeding is often used to compare the group means (male vs. female) on the proposed constructs (Dimitrov, 2006). The fit indices were acceptable (CFI = 0.942, TLI = 0.926, RMSEA 0.066, SRMR = 0.070, BIC = 15179.13). Overall, we found same relationships between coping, Internet-use expectancies, and ICD for male and female participants. For females the direct effect from social aspects to ICD was not significant (β = −0.148, *p* = 0.087) nor for men (β = −0.067, *p* = 0.661), although the effect size was higher descriptively. The effect of psychopathological symptoms to ICD mediated by Internet-use expectancies was only found for women (β = 0.192, *SE* = 0.086, *p* = 0.025). Nevertheless, due to the small sample size for the structural equation models the results should be discussed with caution. The different structural equation models for the female and male sample with the factor loadings and β-weights are represented in Figure 3.

## Discussion

### General discussion of the results

The current study analyzed potential mechanisms such as person's characteristics, coping style, and Internet-related cognitive bias associated with ICD symptoms. The proposed structural equation model was based on the theoretical model of a specific Internet-use disorder by Brand et al. (2016) and an empirical model on generalized Internet addiction by Brand et al. (2014a). Overall, the model with ICD as dependent variable yielded a good fit with the data. The hypothesized model explained 50.8% of the variance of ICD symptoms. The results showed that the relationship between person's characteristics and ICD were partially mediated by coping style and Internet-use expectancies. Furthermore, a direct effect of social aspects such as social loneliness and perceived social support to ICD symptoms was found.

At first, we calculated bivariate correlation between all variables and the s-IAT-communication score, which were significant. This is in line with previous research on ICD. The findings also confirm the hypothesis, that stress vulnerability and self-efficacy correlate with ICD (for the first time).

Second, the hypothesized structural equation model was analyzed. The study found that social aspects play a central role in ICD. High social loneliness and less perceived social support predicted ICD symptoms. Persons who perceive themselves as socially lonely and less socially supported experience more negative consequences due to their online communication behavior, which is in line with previous research (Baker and Oswald, 2010; De Cock et al., 2013; Omar and Subramanian, 2013; Song et al., 2014). The individuals who selected online communication applications as their main online activity seem to gratify social needs online more than in real-life situations (Song et al., 2014). This indicates that online communication applications fulfill a social function and possibly compensate perceived real-life deficits, which seem to be an essential mechanism for problematic communication behavior (Kim et al., 2009; Yadav et al., 2013; Huang et al., 2014). Interestingly, this effect was not mediated by coping strategies or expectancies regarding the helpfulness of the Internet for solving problems or escaping from reality. Hence, the experienced gratification or the compensation of social deficits, which lead to an excessive use of Internet, describes a direct effect without an impact of further cognitive biases.

The current study aimed at identifying mediation effects and at checking the results with previous empirical findings regarding the mechanisms of a generalized Internet addiction (Brand et al., 2014a). There was neither a direct nor a mediated effect of social aspects on a general Internet addiction. Consequently, it can be assumed that the addictive use of Facebook, WhatsApp, or Twitter is associated with social real-life deficits, like perceived social loneliness and less perceived social support. This is not the case for a general overuse of the Internet when no specific application is preferred. Therefore, the preference of online communication applications as a safe, anonymous, controlled environment for communication is associated with less integration in real-life social networks, which is supposed to lead to a dysfunctional use.

The study also showed that dysfunctional coping style and Internet-use expectancies are significant predictors of ICD, which is consistent with other studies about predictors of Internet addiction (Tonioni et al., 2012; Turel and Serenko, 2012; Xu et al., 2012; Tang et al., 2013; Brand et al., 2014a; Kardefelt-Winther, 2014; Lee et al., 2015). Individuals with high expectancies toward the Internet as helpful tool to distract from annoying duties or to experience pleasure as well as with dysfunctional coping strategies such as denial or behavioral disengagement have a higher risk to develop an ICD. The relevance of psychopathological symptoms like social anxiety and depression for an ICD is supported by the suggested model and compatible with other research on the relationship between psychopathological aspects and SNS use (De Cock et al., 2013; Panek et al., 2013; Hong et al., 2014; Bhagat, 2015; Bodroza and Jovanovic, 2015; Laconi et al., 2015; Moreau et al., 2015; Guedes et al., 2016). The effect of psychopathological symptoms to ICD was mediated by the Internet-use expectancies which is consistent with the study by Wegmann et al. (2015). Persons with depressive symptoms, social anxiety, and the expectancies toward the Internet as a helpful tool for escaping from negative feelings and for gratifying social needs, have a higher risk to develop a problematic use of online communication services (Wegmann et al., 2015). Similar to the psychopathological symptoms, the effect of personality aspects like self-esteem, self-efficacy, and stress vulnerability to ICD were mediated by specific cognitions, in this case a dysfunctional coping style. Low self-esteem, self-efficacy, and higher stress vulnerability leads to the denial or problems, substance use, and behavioral disengagement. These individuals have no further strategies to cope with low self-esteem or feelings of loneliness or depression. This association could influence individuals to go online in order to escape from real life problems. Former research already indicated the relationship between self-esteem and the preference for online communication (Chak and Leung, 2004; Steinfield et al., 2008; Panek et al., 2013; Bhagat, 2015; Laconi et al., 2015; Guedes et al., 2016). Consistent with the theoretical approach by Brand et al. (2016), it is assumed that individuals with higher stress vulnerability and deficits concerning their self-confidence in combination with dysfunctional/impulsive coping strategies have a higher need for mood regulation (Whang et al., 2003; Tonioni et al., 2014; Brand et al., 2016). The interaction between these person's characteristics and the individual way to react to difficult situations could result in the use of the “first-choice”-application, i.e., communication applications, in which individuals communicate with others. This behavior can be a very helpful strategy given that individuals discuss their problems with others online. On the other hand, this behavior could be problematic if other problem-solving strategies are neglected and real-life contact is ignored, which could result in higher social isolation. The results indicate that real-life problem-solving strategies play an important role online as well. Conveying functional coping strategies, such as active coping, seems to be an essential preventive mechanism in decreasing the risk to use the Internet or the “first-choice”-application as a dysfunctional coping strategy (Kardefelt-Winther, 2014).

Controlling the results after searching for gender bias, we found some differences in the results for men and women. The results merely revealed that the use of online communication applications when feeling lonely or the perception of less social support was more distinctive for women. Some differences between male and female participants for different Internet-use disorders or SNS use patterns were reported previously (Ko et al., 2005; Meerkerk et al., 2006; Kuss and Griffiths, 2011a; Laconi et al., 2015). Ang (2017) for example, emphasized that females with a stronger Internet habit are more likely to engage in online communication than male participants. Possible differences for ICD have to be investigate in further studies.

In summary, the findings are in line with the theoretical model of Internet-use disorder (Brand et al., 2016) indicating that the relationship between person's characteristics and symptoms of an Internet-use disorder are mediated by specific cognitions. Additionally, the mediation effects which were found in the course of this study have already been supposed for a generalized Internet addiction (Brand et al., 2014a) and cybersex addiction (Laier and Brand, 2014). Nevertheless, the relevance of individual aspects like psychopathological, personality, and social aspects differs. While personality aspects and psychopathological symptoms were mediated by cognitive dimensions assessing a generalized Internet addiction and ICD, social cognitions did not play a role in the development and maintenance of generalized overuse of the Internet. In the current study, social aspects had a direct effect to symptoms of ICD.

Consequently, the current study emphasizes convergent and divergent mechanisms of different forms of Internet-use disorders, as shown by Montag et al. (2015), Laconi et al. (2015), Pawlikowski et al. (2014), and Wang C. W. et al. (2015). While there seems to be an overlap between potential mechanisms of a general overuse of Internet and online communication behavior, evidence was found that allows to differentiate between specific Internet-use disorders. Therefore, it could be concluded that generalized Internet addiction and ICD share common mechanisms but are not synonymous (Hormes et al., 2015). Some investigations show growing evidence that suggests similarities between excessive use of Internet-communication applications and further behavioral addictions. These studies illustrate the relevance of reinforcement mechanisms as well as evidence for several diagnostic criteria, which emphasizes the own construct of an ICD (Kuss and Griffiths, 2011b; Andreassen and Pallesen, 2014; Hormes et al., 2015).

A main conclusion is that the theoretical model of Internet-use disorder (Brand et al., 2014b) could be transferred to ICD, similar to the cybersex addiction case (Laier and Brand, 2014). The modification of this theoretical model into a specific Internet-use disorder, which emphasizes the use of specific, preferred applications, could facilitate the understanding of individual mechanisms. The modified model for an ICD should focus on the role of social aspects and the assumption that persons with perceived social deficits use online communication applications to compensate these deficits directly. This is in contrast with further person's characteristics, which are mediated by specific cognitions. Additionally, the empirical model of the current study should be controlled for other forms like Internet-gaming disorder, Internet-pornography-use disorder, or pathological online buying behavior. For Internet-gaming disorder, individuals could also use the function to communicate online and stay in contact with other gamers while gaming. Consequently, in this case, the potential role of social aspects needs to be discussed as well.

### Limitations

Finally, there are some limitations to be mentioned. First, the study is based on an online survey in a non-clinical sample. Although the data was carefully controlled and removed participants, which answered the questionnaires in an excessively long or short time, we could not exclude potential bias in the data due to the relationship between the online environment of the survey and its content. Second, the Brief COPE by Carver (1997) showed low reliability, which is still comparable to former studies (Carver, 1997; Brand et al., 2014a). However, future studies should consider using another questionnaire or controlling the data and the subscales concerning their reliability. However, we used these subscales to model coping as latent dimension, which means that the effects in the structural equation model were free of measurement errors, although the reliability of the single scales measuring coping were not optimal. Regarding the discussion about the common method bias, a strength of the current study is the heterogeneity of the Likert scales. Podsakoff et al. (2003) emphasize that using common scale formats would refer to artificial covariation. They recommend the use of different scales and constructs to enhance variances and to reduce the common method bias. Thirdly, in the current study the term “Internet-communication application” or “online communication applications” was used. Since this term includes a broad range of different technologies, the effect of the different technologies may be addressed in further research. Nevertheless, to limit this issue, all participants of the study have been given a clear definition, of the term “Internet-communication applications.” Additionally, variables such as self-efficacy could be specified for the dependent variables and the underlying mechanisms, for example using Internet self-efficacy or self-efficacy toward these different online communication applications.

### Future research

Future research should investigate direct convergent and divergent mechanisms of different types of Internet-use disorders. In the current study a structural equation model was used and the results were compared to other empirical findings in the literature. However, a direct empirical comparison should expand our knowledge about the different contributions of social aspects in the development and maintenance of different types of Internet-use disorders.

## Author contributions

EW: Wrote the first draft of the paper, supervised the preparation of the manuscript, and contributed intellectual and practical work to the manuscript; MB: Edited the draft, revised it critically, and contributed intellectually and practically to the manuscript. Both authors finally approved the manuscript. Both authors are accountable for all aspects of the work.

### Conflict of interest statement

The authors declare that the research was conducted in the absence of any commercial or financial relationships that could be construed as a potential conflict of interest.
